# Supplementary material for: In Silico Approach for the Evaluation of the Potential Antiviral Activity of Extra Virgin Olive Oil (EVOO) Bioactive Constituents Oleuropein and Oleocanthal on Spike Therapeutic Drug Target of SARS-CoV-2
Source: Molecules. 2022 Nov 4;27(21):7572. doi: 10.3390/molecules27217572 (PMC9655607; doi:10.3390/molecules27217572)
Supplement: Supplementary file 1 [file molecules-27-07572-s001.zip › molecules-1961753-supplementary.pdf]

molecules

Article

In Silico Approach for the Evaluation of the Potential Antiviral Activity of Extra Virgin Olive Oil (EVOO) Bioactive Constituents Oleuropein and Oleocanthal on Spike Therapeutic Drug Target of SARS-CoV-2

Elena G. Geromichalou [1,*], and George D Geromichalos [2,*]

[1] Laboratory of Pharmacology, Medical School, National and Kapodistrian University of Athens, 75 Mikras Asias Street, 11527 Athens, Greece
[2] Department of General and Inorganic Chemistry, Faculty of Chemistry, Aristotle University of Thessaloniki, 54124 Thessaloniki, Greece
* Correspondence: elena_geromich@outlook.com (E.G.G.); gerom@chem.auth.gr or geromchem@yahoo.gr (G.D.G.)

Supplementary Materials

Index

Content		Page
**S1.**	**Computational methods**	3
S1.1.	*In silico molecular docking*	3
**S2.**	***In silico* molecular docking studies**	9
S2.1.	*Binding of OC in the crystal structure of SARS–CoV–2 full–length model of the Spike (S) trimeric protein in the open conformation state (one RBD–up)*	11
S2.2.	*Binding of OEU and OC on the crystal structure of wild–type (wt) SARS–CoV–2 full–length model of the Spike (S) protein in the closed conformation state (all RBDs–down)*	12
S2.3	*D614G mt open Spike protein (one RBD–up) (7KDL)*	14
S2.4	*Omicron BA.3 mt RBD of S protein (7XIZ)*	14
S2.5	*Wt open Spike protein/ACE2 complex (7KJ2)*	14
S2.6	*Omicron BA.2 mt Spike protein/ACE2 complex (two and three ACE2 bound) (7XO7 and 7XO8)*	14
S2.7	*Wt full–length S protein's RBD/ACE2 complex (from 6M17)*	14
S2.8	*Wt S proteins' RBD/ACE2 complex (6VW1)*	15
S2.9	*Delta and Kappa S proteins' RBD/ACE2 complex (7V8B and 7V87)*	15
S2.10	*Omicron BA.1 and BA.2 mt S proteins' RBD/ACE2 complex (PDB ascension N's 7WPB and 7XO9)*	15
S2.11	*Omicron BA.2 mt S proteins' RBD/ACE2 complex (ascension Nr 7ZF7)*	16
S2.12	*N501Y mt RBD in complex with COVOX–269 Fab (7NEG)*	16
**References**		17

Scheme S1. Flow chart of the computational procedure. 8

Figure S1. The enantiomer molecular structures of EVOO constituent Oleuropein (OEU), 4S6S and 4S6R, indicating the chiral center of each one, rendered in ball–and–stick model colored according to atom type in slate blue and violet purple C atoms. Hydrogen atoms are omitted. The final structure was ray–traced and illustrated with the aid of PyMol Molecular Graphics System (Heteroatom color–code: O in red). 9

Figure S2. The enantiomer molecular structures of EVOO constituent Oleocanthal (OC), (S)-(+) and (R)-(–), indicating the chiral center of each one, rendered in ball–and–stick model colored according to atom type in slate blue and violet purple C atoms. Hydrogen atoms are illustrated in line mode. The final structure was ray–traced and illustrated with the aid of PyMol Molecular Graphics System (Heteroatom color–code: O in red). 10

Figure S3. A close–up view of the binding site mapping architecture of the best binding pose of OC in the crystal structure of SARS–CoV–2 full–length model of the Spike (S) trimeric protein in the open conformation state (one RBD–up). Target protein is depicted in cartoon colored by chain. OC rendered in stick mode and colored according to atom type in yellow orange C atoms is stabilized at the interface between the NTD (14–305) (part of the S1 of A) of protomer A and the RBD 11

domain of protomer C (in purple and orange color, respectively). Selected contacting amino acid residues belonging to protomers A and C of the binding pocket are rendered in stick model and colored according to chain. Binding contacts are shown as dotted yellow lines. Hydrogen atoms are omitted for shake of clarity. Heteroatom color–code: O: red. The final structure was ray–traced and illustrated with the aid of PyMol Molecular Graphics Systems.

Figure S4. Docking pose orientation of best bound OEU and OC molecules, on the crystal structure of wild–type (wt) SARS–CoV–2 full–length model of the Spike (S) protein in the closed conformation state (all RBDs–down), based on PDB: 6VXX and embedded in a lipid bilayer mimicking the composition of the endoplasmic reticulum–Golgi intermediate compartment after molecular dynamics simulation. Target trimeric wt S protein is illustrated as cartoon colored by chain in orange, chocolate, and split pea green for each of the 3 protomers (a, b, and c, respectively). OEU and OC are rendered in sphere mode and colored according to atom type in white and hot pink C atoms, respectively. OEU is located adjacent to S1/S2 furin cleavage site to S2′ (686-815) and in contact with the native D614 residue rendered in light pink sphere mode. Color code used for lipid tails (surface representation): POPC, POPE, POPI, POPS, and cholesterol in cyan. P atoms of the lipid heads and cholesterol's O3 atoms are highlighted in red. N–linked glycans (NAG moieties) are omitted from the structure for shake of clarity. Molecular docking simulations were performed individually. Hydrogen atoms are omitted from both molecules and sugar molecules glycosylating the protein are hided for shake of clarity. Heteroatom color–code: O: red. The final structure was ray–traced and illustrated with the aid of PyMol Molecular Graphics Systems. 12

Figure S5. A close–up view of the binding site mapping architecture of the best binding pose of OEU in the crystal structure of SARS–CoV–2 full–length model of the Spike (S) trimeric protein in the closed conformation state (all RBDs–down). Target protein (part of protomer B) is depicted in opaque surface colored in split pea green with additional depiction of selected contacting amino acid residues belonging to protomer B of the binding pocket highlighted in orange. OEU, rendered in stick mode and colored according to atom type in white C atoms, is in close contact to the native D614 residue highlighted in light pink on the surface. Binding contacts are shown as dotted yellow lines. Hydrogen atoms are omitted for shake of clarity. Heteroatom color–code: O: red. The final structure was ray–traced and illustrated with the aid of PyMol Molecular Graphics Systems. 13

S1. Computational methods

S1.1. In silico molecular docking

The *in silico* predictive tools that have been employed to study the interaction of OEU and OC with the selected macromolecules, are Schrödinger, BIOVIA Discovery Studio 2016, ChemBio3D Ultra v. 14.0.0.117 software suite, Spartan′ 14 and PyMol molecular modeling software. The structures of OEU and OC were retrieved by PubChem chemical information resource library at the U.S. National Center for Biotechnology Information (NCBI) (https://pubchem.ncbi.nlm.nih.gov) (OEU: CID_35281544 and OC: CID_11652416). 3D conformer of each structure was built with the aid of ChemBio3D Ultra v. 14.0.0.117 software suite (CambridgeSoft Corporation) and after structure optimization through energy minimization (MM2 force field method), pdb files of each structure were

generated. The best, most stable (lowest energy) conformation of the molecular model of the complexes were detected by geometrical optimization in the gas phase, as implemented in the Spartan '14 Molecular Modeling program suite (Spartan '14 v.1.1.4, Wavefunction Inc., Irvine, CA, USA; www.wavefun.com.). The structures were initially optimized (via energy minimization) by conformational search using the Monte Carlo method with the MMFF94 molecular mechanics model, included in the Spartan'14 program suite. Geometry optimization (leading to the most stable conformer with the lowest energy) was accomplished *via* quantum-chemical calculations by utilizing Density functional theory (DFT) computations at B3LYP level of theory with 6-31G*(d,p) basis set to describe the accurate structural and electronic properties of the compounds, implemented by Spartan' 14 program suite.

Molecular docking calculations were carried out on the crystal structure of the following SARS–CoV–2 target proteins:

1) three–dimensional structure of the full-length model of the spike protein in open conformation derived by extensive massive all–atom molecular dynamics (MD) simulations of the glycosylated full–length model of the SARS–CoV–2 spike protein embedded in a realistic compartment membrane/aqueous environment encompassing ~1.7 million atoms [1]. The structural models of wt full–length spike protein in open conformation state (based on the PDB ID: 6VSB) [2] and in closed conformation state (based on the PDB ID: 6VXX) [3] were downloaded from the Amaro lab (https://amarolab.ucsd.edu/covid19.php),

2) spike (S) protein in open conformation state (one RBD–up) (PDB ID 6VYB refined at 3.2 Å resolution) [3],

3) spike (S) protein in open conformation state (two RBDs–up) (PDB ID 7A93 refined at 5.9 Å resolution) [4],

4) spike (S) protein in closed conformation state (Receptor Binding Domain, RBD in down position (three RBDs–down) (PDB ID 6VXX refined at 2.80 Å resolution) [3],

5) spike (S) protein in open conformation state (one RBD–up conformation with D614G mutation, PDB ID 7KDL refined at 2.96 Å resolution) [5],

6) Alpha mt variant 501Y.V1 (B.1.1.7 lineage) spike (S) protein in open conformation state (one RBD–up) (PDB ID 8DLI refined at 2.56 Å resolution) [6],

7) Beta mt variant 501Y.V2 (B.1.351 lineage) spike (S) protein in open conformation state (one RBD–up) (PDB ID 8DLL refined 2.56 Å resolution) [6],

8) Gamma mt variant 209/501Y.V3, 484K.V2 (B.1.1.28 or P1 lineage) spike (S) protein in open conformation state (one RBD–up) (PDB ID 8DLO refined at 2.25 Å resolution) [6],

9) Delta mt variant (B.1.617.2 lineage) spike (S) protein in open conformation state (one RBD–up) (PDB ID 7V7O refined at 2.90 Å resolution) [7],

10) Epsilon mt variant (California B.1.429 lineages) spike (S) protein in open conformation state (one RBD–up) (PDB ID 8DLT refined at 2.40 Å resolution) [6],

11) Kappa mt variant (B.1.617.1 lineage) spike (S) protein in open conformation state (one RBD–up) (PDB ID 7V7E refined at 2.90 Å resolution) [7],

12) Omicron BA.1 mt variant (B.1.1.529.1 sub–lineage) spike (S) protein in open conformation state (one RBD–up) (PDB IDs 7TGW and 7QO7 refined at 3.00 Å and 3.02 Å resolution, respectively) [8]

13) Omicron BA.2 mt variant (B.1.1.529.2 sub–lineage) spike (S) protein in open conformation state (one RBD–up) (PDB ID 7XIW refined at 3.62 Å resolution) [9],
14) Omicron BA.2.13 mt variant spike (S) protein in closed conformation state (all RBDs–down) (PDB ID 7XNR refined at 3.49 Å resolution) [9],
15) Omicron BA.3 mt variant spike (S) protein in closed conformation state (all RBDs–down) (PDB ID 7XIY refined at 3.07 Å resolution) [9],
16) Omicron BA.4 mt variant spike (S) protein in closed conformation state (all RBDs–down) (PDB IDs 7XNQ and 7XNS refined at 3.48 Å resolution) [9],
17) crystal structure of the N501Y mutant RBD domain of SARS–CoV–2 S glycoprotein in complex with COVOX–269 Fab (PDB ID 7NEG refined at 2.19 Å resolution) [10],
18) Omicron BA.3 mt variant RBD of spike (S) protein (PDB ID 7XIZ refined at 3.74 Å resolution) [9],
19) Omicron BA.2 mt variant spike (S) protein in complex with Fab BD55–5840 (PDB ID 7X6A refined at 3.50 Å resolution) [9],
20) Omicron BA.4–5 mt RBD in complex with Beta–27 Fab and C1 nanobody (PDB ID 7ZXU refined at 1.89 Å resolution) [11],
21) Wt spike (S) protein in open (one RBD–up) conformation state complexed with host human Angiotensin Converting Enzyme 2 (hACE2) receptor conformation, PDB ID 7KJ2 refined at 3.60 Å resolution) [12],
22) Omicron BA.2 mt variant spike/ACE2 complex (two ACE2 bound) (PDB ID 7XO7 refined at 3.38 Å resolution) [13],
23) Omicron BA.2 mt variant spike/ACE2 complex (three ACE2 bound) (PDB ID 7XO8 refined at 3.48 Å resolution) [13],
24) Wt full–length S proteins' RBD/ACE2 complex (from 6M17) [1],
25) RBD domain of wt S protein in complex with ACE-2 receptor (PDB ID 6VW1 refined at 2.68 Å resolution) [14],
26) RBD domain of Delta mt variant S protein in complex with ACE2 receptor (PDB ID 7v8b refined at 3.20 Å resolution) [7],
27) RBD domain of Kappa mt variant S protein in complex with ACE2 receptor (PDB ID 7v87 refined at 3.30 Å resolution) [7],
28) RBD domain of Omicron BA.1 mt variant (B.1.1.529 lineage) S protein in complex with ACE2 receptor (PDB ID 7WPB refined at 2.79 Å) [15], and
29) RBD domain of Omicron BA.2 mt variant S protein in complex with ACE2 receptor (PDB IDs 7XO9, refined at 3.00 resolution) [13].
30) RBD domain of Omicron BA.2 mt variant S protein in complex with ACE2 receptor (PDB 7ZF7, refined at 3.46 Å resolution) [16].

All PDB files were obtained from the Brookhaven Protein Data Bank (operated by the Research Collaboratory for Structural Bioinformatics, RCSB) [17-19]. In our studies, molecular docking calculations were performed with Schrödinger modeling suite. In this study we used the accurate GlideScore Extra Precision (XP) in predicting binding affinity. Glide uses Emodel to pick the "best" pose of a ligand (pose selection), and then ranks these best poses against one another with GlideScore. The Schrödinger software suite contains a broad array of computational chemistry tools. In the procedure for molecular docking with the employment of Schrödinger suite (the flow chart of the computational

procedure is depicted in Scheme S1) both compounds were sketched and converted into three–dimensional MOL2 files using Schrödinger Release 2020–3 Maestro Version 11.1 and minimized using LigPrep 3.5 [20] (which can generate a number of structures from each input structure with various ionization states, tautomers, stereochemical characteristics, and ring conformations to eliminate molecules on the basis of various criteria such as molecular weight or specified numbers and types of functional groups with correct chiralities for each successfully processed input structure), and the OPLS3 (**Opt**imized **P**otential for **L**iquid **S**imulations) [21] force field for the optimization, producing the low-energy isomers of the ligands (Schrödinger, http://www.schrodinger.com). Energy minimized 3D molecular structures were generated with the employment of LigPrep run from Maestro utility of the Schrödinger suite. The ligand preparation included 2D–3D conversions, generating variations, correction, verification and optimization of the structures. A preparation of receptor and ligand structures was integrated before the actual docking procedure [22]. The crystal structures of the proteins were prepared using the Protein Preparation Wizard [22], in Schrödinger Suite 2020–3 (Schrödinger, LLC, New York, NY) [23,24]. Protein was prepared by adding the hydrogen atoms, optimizing hydrogen bonds, removing atomic clashes, adding formal charges to the hetero groups and then optimizing at neutral pH. Missing loops and side chains were prepared using Prime version 3.2 [25,26]. Finally, the structure was minimized using OPLS3 force field. Active site of studied proteins was obtained using SiteMap tool (version 3.6, Schrödinger) [27,28], which provides a fast and effective means of identifying potential binding pockets of proteins. SiteMap identifies the character of binding sites using novel search and assesses each site by calculating various properties like size, volume, amino acid exposure, enclosure, contact, hydrophobicity, hydrophilicity and donor/acceptor ratio. Receptor grid was generated around the active site for effective binding using Receptor grid generation in the Glide (version 5.9) application of Maestro. Once the receptor grid is generated, the ligands are docked to the proteins using Glide docking tool of Schrödinger (**G**rid based **Li**gand **D**ocking with **E**nergetics) (version 6.8) [24,29]. Compounds were docked in the binding site of the proteins using Induced Fit Docking (IFD) protocol 2020–3 [30-32]. The ligand interactions are shown in Ligand interaction tool of Maestro (Schrödinger). Waters were deleted with Maestro, the graphical user interface (GUI) of Schrödinger software, prior to docking. Molecular docking studies were carried out for the best fitted compounds to the model, while the final selection criteria were compounds docking scores and the presence of crucial interactions for binding to the studied proteins [33]. The resulting poses were examined manually and the most promising ones were redocked with IFD calculations. Poses that pass the initial screens enter the final stage of the algorithm, which involves evaluation and minimization of a grid approximation to the OPLS–AA nonbonded ligand–receptor interaction energy. Final scoring is then carried out on the energy–minimized poses. By default, Schrödinger's proprietary Glide Score [28] multi–ligand scoring function is used to score the poses. The rescoring was performed to calculate and improve binding energy calculations and accuracy with Prime's Molecular Mechanics–Generalized Born Surface Area (MM–GBSA) protocol using VSGB solvation model [34]. Another strategy is the use of molecular dynamics (MD) simulation to get enhanced conformational sampling of the protein–ligand complex obtained by using docking, and subsequent calculation of the binding energy by averaging the score values for different poses extracted from the trajectory. In this way the stability and dynamical changes of predicted binding conformations could be determined, providing additional insights into time–dependent configurational changes of the structures, which is crucial for correct

prediction of ligand binding and related thermodynamic and kinetic property calculations. Under this approach, the receptor flexibility and the presence of water molecules contribute to a more realistic description of the complex, which could have an influence in binding energy calculations. Nevertheless, due to a great number of docking computations employed in the current study (exceeding 60 in number) and although such an approach would definitely be interesting to explore, an extensive sampling could impose α significant hindrance of the whole procedure since the computational cost of MD would definitely be too computationally expensive and extremely time–consuming to perform. It is clearly accepted that scoring functions, which come with popular docking programs, are not good predictors (when rescoring process is not performed). In other words, common molecular docking programs should not be used for accurately predicting binding affinity energy values.

Both compounds showed good docking scores reflecting drug–binding affinities with the studied proteins.

It is of paramount importance the incorporation of target protein flexibility in the docking procedure compared to conventional rigid receptor docking methods. On the other hand, molecular docking simulations with flexible proteins have to deal with the increased amount of computational complexity that is introduced by protein flexibility. Nevertheless, by adopting the Glide docking tool of Schrödinger software suite, partial protein flexibility may be encountered. Glide evaluate PDB temperature factors, align binding sites, and run IFD protocol for side chain conformational changes and loop refinement. IFD generates binding poses for targets where there is suspected conformational flexibility, in both the ligand and receptor, which is crucial for accurate docking of the ligand. It generates new complexes by optimizing active–site side chains in the presence of possible ligand poses. This protocol uses Glide to sample ligand binding modes and Prime to sample protein conformation flexibility and is a useful tool to predict active site geometries with minimal expense. IFD primarily explores side–chain motion, though the receptor backbone can move a bit during the Prime optimization stage, which includes both side–chain rearrangement and minimization of the active site. Nevertheless, larger backbone motions are not encountered, since IFD only allows local movements of some selected residues in the active site and its accuracy largely depends on the precision of Glide. It is understandable that the protein conformations produced by MD simulations perform better than those obtained based on the original crystal structures. It should also be noted that the protein flexibility may be imperative when it seems to have a conspicuous influence on docking accuracy, a fact that cannot be validated for the structures in use. The procedure may cause some perturbation in the reported stability of the docking ligand–protein complexes since traditional MD simulations would be necessary. Nevertheless, due to large number of docking computations (over 60 in number, including 34 target proteins) it seemed almost impossible to successfully perform this large number of highly time–demanding MD computations.

PyMol Molecular Graphics System (Schrödinger, LLC. version 2.3.5, www.pymol.org) [35] was used to visualize the molecules and analyze the results of the docking and to construct the molecular models. BIOVIA Discovery Studio 2016 (BIOVIA, Discovery Studio v 16.1.0.15350, San Diego: Dassault Systèmes, 2016) was employed for the visualization of the binding interactions between OEU and OC molecules on the crystal structure of the target proteins.

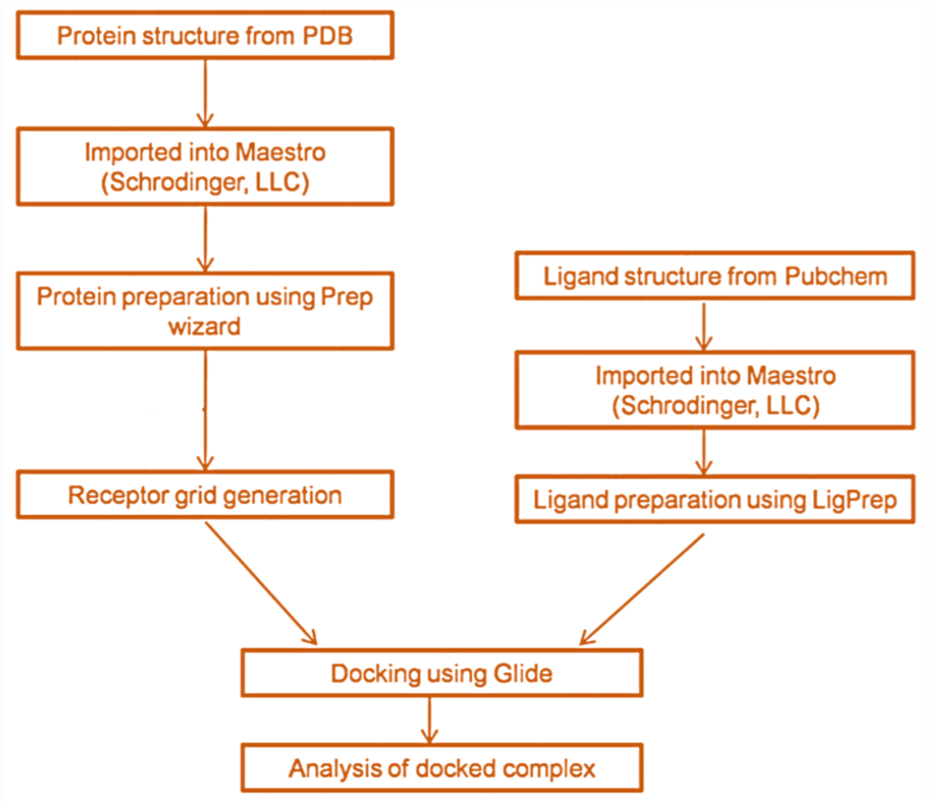

Scheme S1. Flow chart of the computational procedure.

S2. *In silico* molecular docking studies

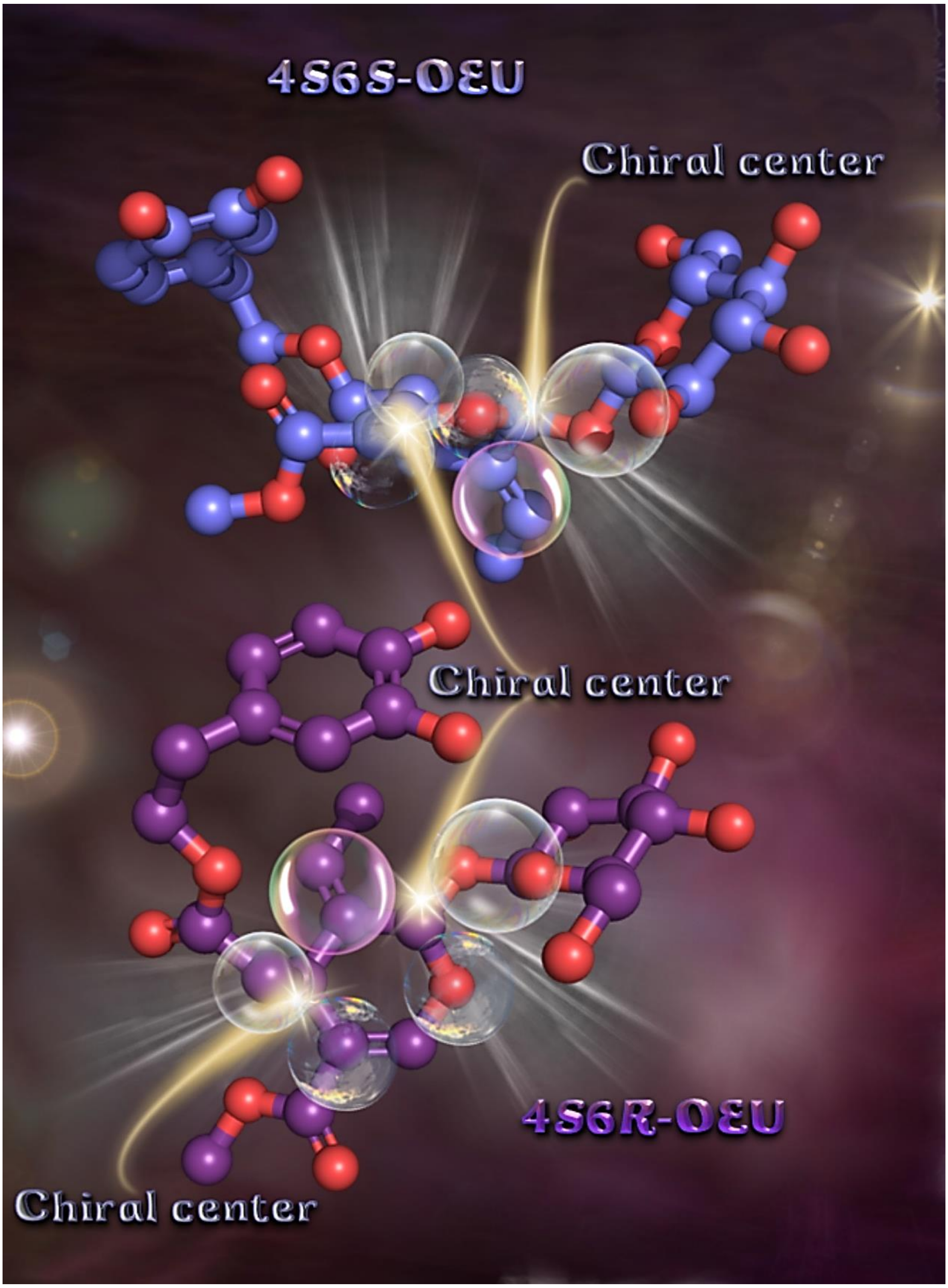

Figure S1. The enantiomer molecular structures of EVOO constituent Oleuropein (OEU), 4S6S and 4S6R, indicating the chiral center of each one, rendered in ball–and–stick model colored according to atom type in slate blue and violet purple C atoms. Hydrogen atoms are omitted. The final structure was ray–traced and illustrated with the aid of PyMol Molecular Graphics System (Heteroatom color–code: O in red).

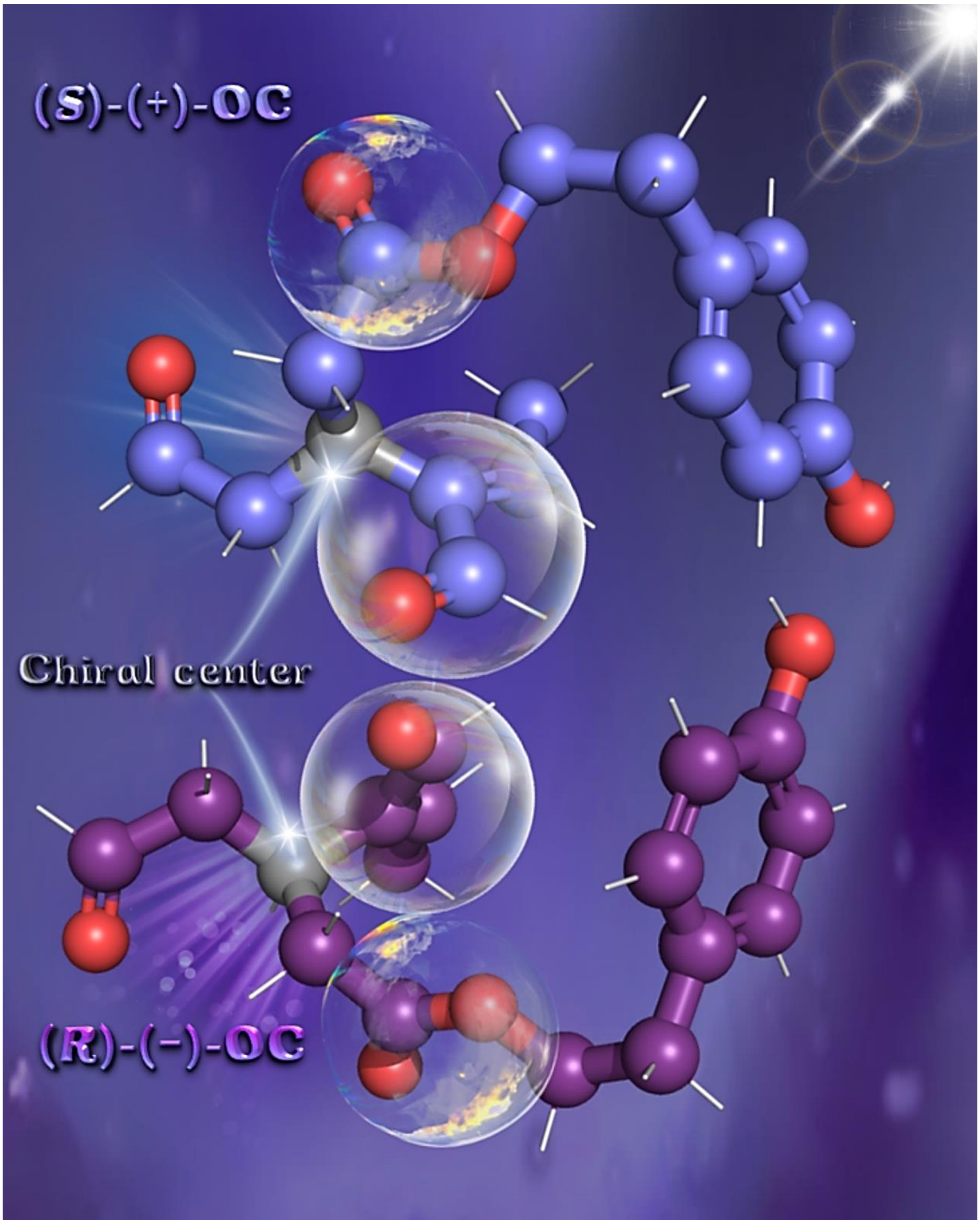

Figure S2. The enantiomer molecular structures of EVOO constituent Oleocanthal (OC), (S)-(+) and (R)-(−), indicating the chiral center of each one, rendered in ball–and–stick model colored according to atom type in slate blue and violet purple C atoms. Hydrogen atoms are illustrated in line mode. The final structure was ray–traced and illustrated with the aid of PyMol Molecular Graphics System (Heteroatom color–code: O in red).

S2.1. Binding of OC in the crystal structure of SARS–CoV–2 full–length model of the Spike (S) trimeric protein in the open conformation state (one RBD–up)

OC interactions involve binding with T114 (2.9, 3.1 Å), Q115 (2.0, 2.4, 2.7 Å), N165 (3.3 Å), T167 (2.1, 2.3 Å), G232 (3.9 Å), I233 (2.0, 3.1 Å), and N234 (2.8, 2.9 Å) residues of protomer a, and with N354 (2.7 Å), R355 (2.8 Å), K356 (3.1, 3.8 Å), R357 (2.9 Å), and R466 (2.4, 2.5, 3.1 Å) residues of protomer c. OEU interactions involve binding with G548 (3.5 Å), T549 (3.2 Å), A570 (2.4 Å), T572 (2.5 Å), T573 (2.3 Å), I587 (3.9 Å), and P589 (2.6 Å) residues of protomer b (deep teal color), and also with M740 (2.7 Å), Y741 (3.1, 3.3 Å), G744 (2.5 Å), D745 (3.5 Å), F855 (4.0 Å), N856 (2.7, 3.0, 3.6 Å), V963 (2.6, 2.9 Å), L966 (3.9 Å), S967 (3.0, 3.5 Å), and N978 (2.8 Å) residues of protomer c (orange color).

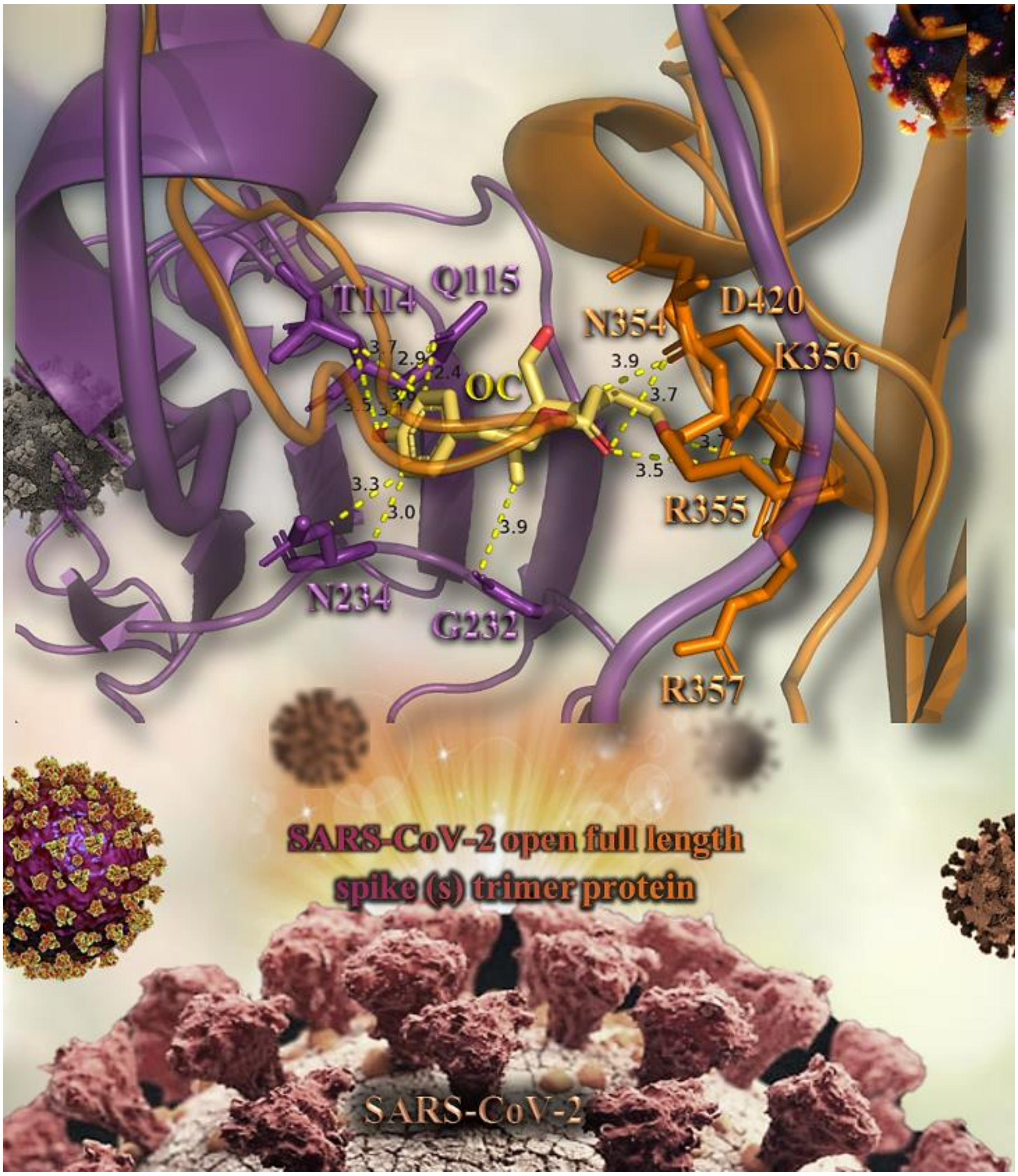

Figure S3. A close–up view of the binding site mapping architecture of the best binding pose of OC in the crystal structure of SARS–CoV–2 full–length model of the Spike (S) trimeric protein in the open conformation state (one RBD–up). Target protein is depicted in cartoon colored by chain. OC rendered in stick mode and colored according to atom type in yellow orange C atoms is stabilized at the interface between the NTD (14–305) (part of the S1

of A) of protomer A and the RBD domain of protomer C (in purple and orange color, respectively). Selected contacting amino acid residues belonging to protomers A and C of the binding pocket are rendered in stick model and colored according to chain. Binding contacts are shown as dotted yellow lines. Hydrogen atoms are omitted for shake of clarity. Heteroatom color–code: O: red. The final structure was ray–traced and illustrated with the aid of PyMol Molecular Graphics Systems.

S2.2. Binding of OEU and OC on the crystal structure of wild–type (wt) SARS–CoV–2 full–length model of the Spike (S) protein in the closed conformation state (all RBDs–down)

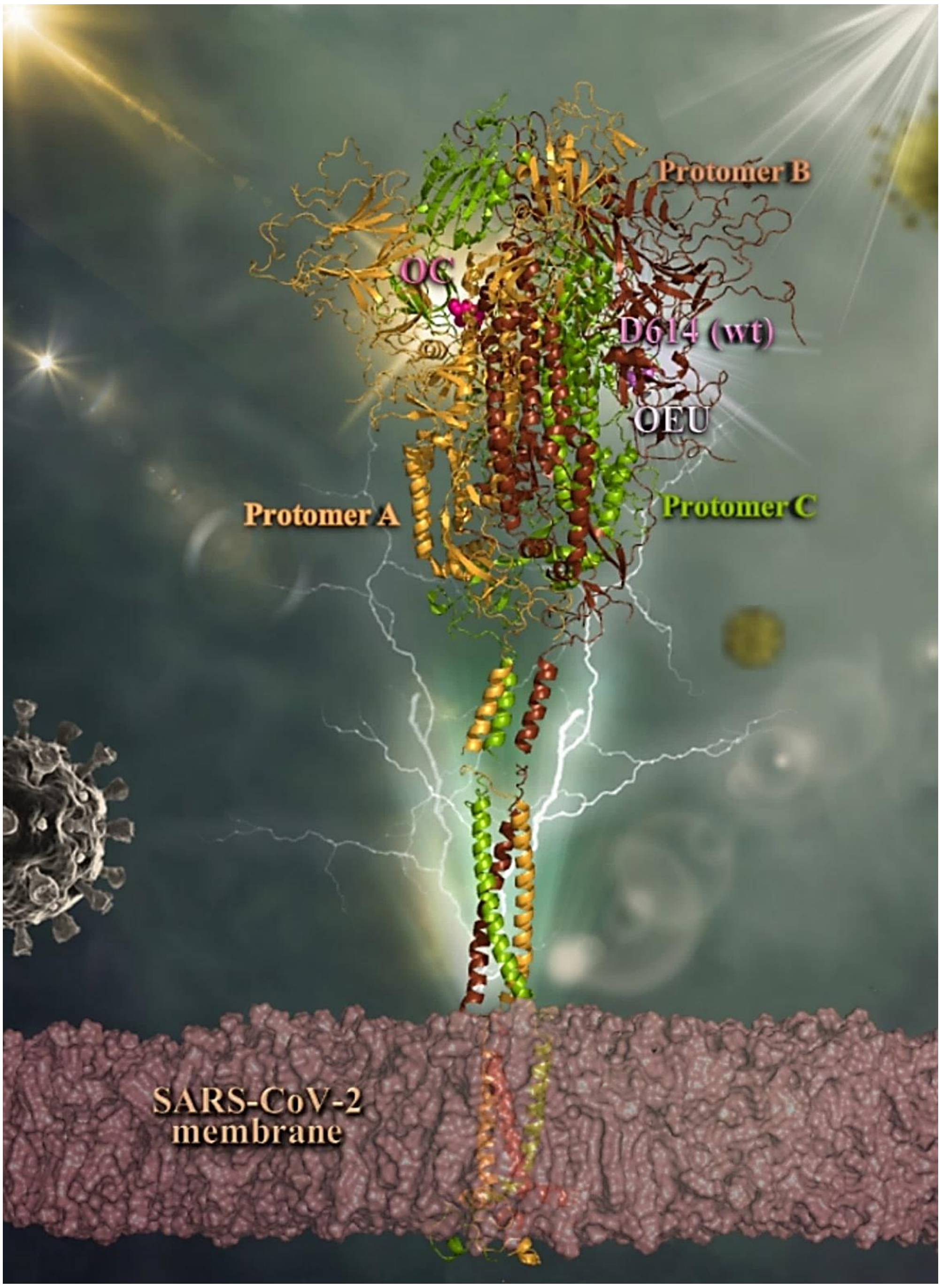

Figure S4. Docking pose orientation of best bound OEU and OC molecules, on the crystal structure of wild–type (wt) SARS–CoV–2 full–length model of the Spike (S) protein in the closed conformation state (all RBDs–down), based on PDB: 6VXX and embedded in a lipid bilayer mimicking the composition of the endoplasmic reticulum–Golgi intermediate compartment after molecular dynamics simulation. Target trimeric wt S protein is illustrated

as cartoon colored by chain in orange, chocolate, and split pea green for each of the 3 protomers (a, b, and c, respectively). OEU and OC are rendered in sphere mode and colored according to atom type in white and hot pink C atoms, respectively. OEU is located adjacent to S1/S2 furin cleavage site to S2′ (686-815) and in contact with the native D614 residue rendered in light pink sphere mode. Color code used for lipid tails (surface representation): POPC, POPE, POPI, POPS, and cholesterol in cyan. P atoms of the lipid heads and cholesterol's O3 atoms are highlighted in red. N–linked glycans (NAG moieties) are omitted from the structure for shake of clarity. Molecular docking simulations were performed individually. Hydrogen atoms are omitted from both molecules and sugar molecules glycosylating the protein are hided for shake of clarity. Heteroatom color–code: O: red. The final structure was ray–traced and illustrated with the aid of PyMol Molecular Graphics Systems.

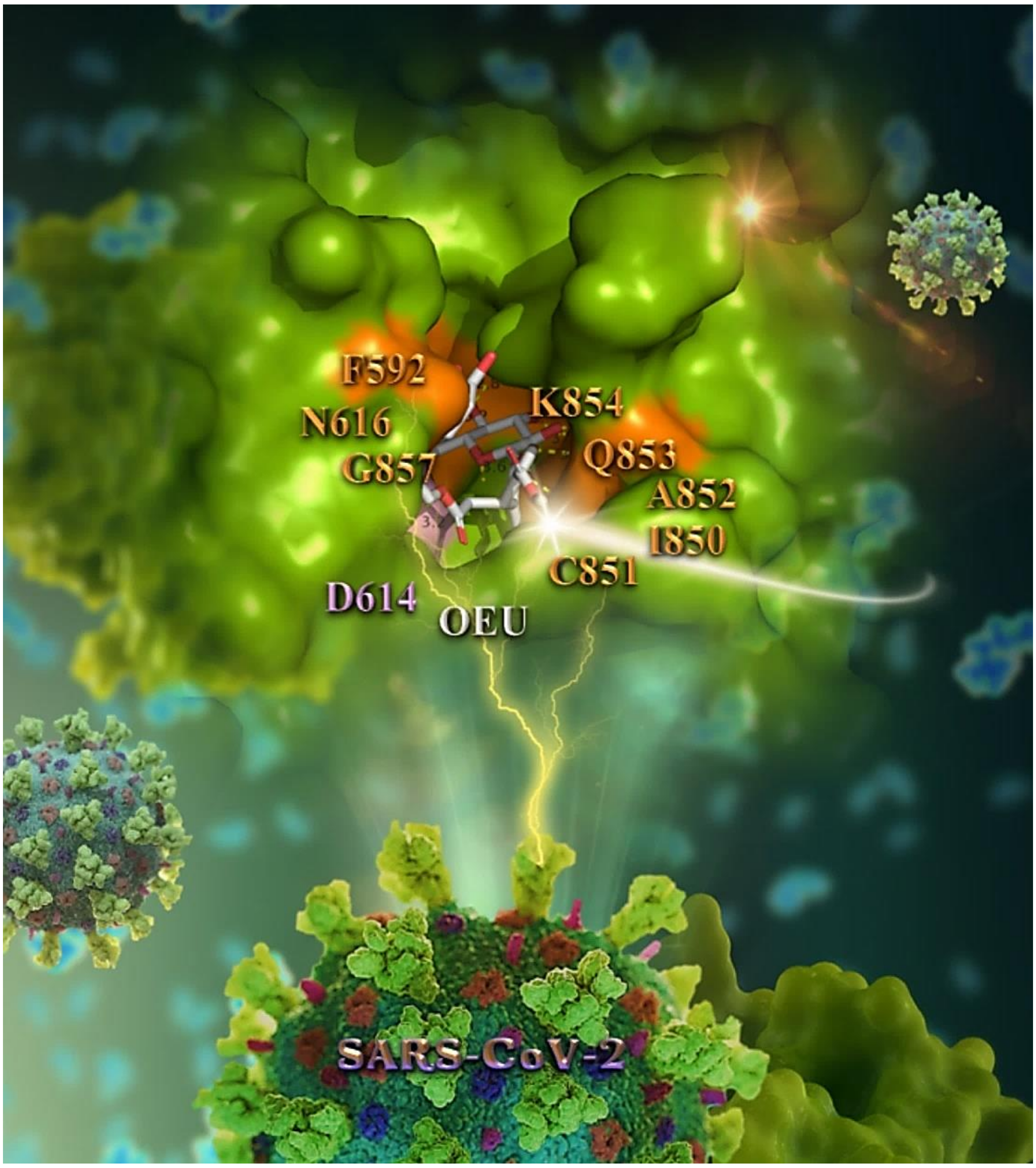

Figure S5. A close–up view of the binding site mapping architecture of the best binding pose of OEU in the crystal structure of SARS–CoV–2 full–length model of the Spike (S) trimeric protein in the closed conformation state (all RBDs–down). Target protein (part of protomer B) is depicted in opaque surface colored in split pea green with additional depiction of selected contacting amino acid residues belonging to protomer B of the binding pocket highlighted in orange. OEU, rendered in stick mode and colored according to atom type in white C atoms, is in close contact to the native D614 residue highlighted in light pink on the surface. Binding contacts are shown as dotted yellow lines. Hydrogen atoms are omitted for shake of clarity. Heteroatom color–code: O: red. The final structure was ray–traced and illustrated with the aid of PyMol Molecular Graphics Systems.

S2.3. D614G mt open Spike protein (one RBD–up) (7KDL)

Both OEU and OC are stabilized inside the RBD in the "up" position exactly at the same place sharing a number of contacts including residues F338/Cε2 (π–polar, 3.8 Å, π–alkyl, 3.0 Å), F342/Cζ (π–alkyl, 3.5 Å), Y365/Cβ (polar, 2.5 Å), L368/Cβ (polar, 3.6 Å), F377/Cβ (π–alkyl, 3.4 Å), K378/O (H–bond, 3.2 Å, **P384**/Cγ (polar, 2.9 Å), L390/C (polar, 3.0 Å), **F392**/Cβ (polar, 3.2 Å), A397/Cβ (Hph, 3.4 Å), **T430**/O (H–bond, 3.2 Å), **C432**/Sγ (polar, 3.1 Å), V433/O (H–bond, 3.4 Å), I434/Cβ (π–alkyl, 3.1 Å, π–alkyl, 3.7 Å), V511/O (polar, 3.0 Å), **L513**/NH,Cβ (H–bond, 2.9 Å, Hph, 3.3 Å), and **F515**/Cβ (polar, 3.0 Å) for OEU, and F338/Cε2 (π–polar, 3.9 Å), Y365/Cβ (Hph, 3.7 Å), **P384**/Cβ (polar, 3.4 Å), L387/Cβ (Hph, 3.9 Å), **F392**/Cβ (Hph, 3.7 Å), **T430**/O (H–bond, 3.4 Å), **C432**/O, Sγ (π–polar, 2.9 Å, H–bond, 3.5 Å), **C432**/N, Cβ (polar, 3.9 Å, π–alkyl, 3.3 Å), **L513**/O (H–bond, 3.4 Å), L513/Cβ (π–alkyl, 3.3 Å), and **F515**/N, Cα (H–bond, 3.5 Å, Hph, 3.1 Å), for OC.

S2.4. Omicron BA.3 mt RBD of S protein (7XIZ)

Both OEU and OC are shown to be stabilized at the interface between the RBD–core and the RBM motif. OEU is predicted to be anchored in the vicinity of the base of a twisted five–stranded antiparallel sheet bundle (β1, β2, β3, β4, and β7) interacting with residues F342/O (polar, 3.4 Å), N343/O (π–polar, 3.8 Å), A344/O (π–polar, 3.4 Å), T345/O (H–bond, 2.5 Å, π–polar, 3.0 Å), F371/O (polar, 3.7 Å), P373/Cδ (Hph, 2.8 Å), W436/O (π–polar, 3.7 Å), N437/O (H–bond, 2.3 Å), N439/Oδ1 (H–bond, 3.5 Å), K440/Nζ (H–bond, 2.3 Å), L441/Cδ1 (Hph, 3.3 Å), and R509/Nη1 (H–bond, 2.6 Å). Binding contacts of OC include the residues N394/Oδ1 (H–bond, 2.5 Å), R355/Nη2 (H–bond, 3.9 Å), N394/Oδ1 (H–bond, 2.5 Å), P426/C (π–alkyl, 3.9 Å), D428/Oδ1 (H–bond, 3.0 Å, π–anion, 2.8 Å), F429/N (polar, 3.8 Å), T430/N (polar, 2.7 Å), F464/Cε2 (π–polar, 2.5 Å), E516/Oε1 (H–bond, 3.7 Å), and F515/O (polar, 2.1 Å).

S2.5. Wt open Spike protein/ACE2 complex (7KJ2)

The docking procedure predicts the formation of a variety of interactions between OEU and the amino acid residues Ala A520/O (H–bond, 2.1 Å, π–polar, 3.5 Å), His H519/Nδ1 (H–bond, 2.9 Å), His H519 (π–polar, 3.4 Å), Arg R567/Nη1 (H–bond, 3.4 Å), Asp D571/O (H–bond, 2.5 Å), Thr T573/N, Oγ1 (H–bond, 2.9, 3.2 Å), Leu L517/O, Cδ1 (π–polar, 3.5 Å, Hph, 2.7 Å), Cys C391/O (polar, 3.2 Å), Cys C391 (π–polar, 3.5 Å), Thr T393/ N, Cγ2 (π–polar, 3.8 Å, π–alkyl, 4.1 Å), Asn N544/O (polar, 3.1 Å), Gly G545/NH, C (H–bond, 3.3 Å, Hph, 2.8 Å), Leu L546/Cδ1, Cα (Hph, 2.2, 3.3 Å, polar 2.3 Å), and Thr T547/O, Oγ1 (H–bond, 3.8 Å, polar, 3.8 Å) of protomer a, and Val V976/Cγ2 (Hph, 2.7 Å), Asn N978/O (polar, 2.9 Å), Asp D979/Oδ1, Cα, N (H–bond, 3.9 Å, polar, 2.3 Å, Hph, 2.6 Å, polar, 3.0 Å), and Ser S982/Oγ (polar, 3.1 Å) of protomer b. OC and OEU are sharing common binding contacts since they are anchored exactly at the same place.

S2.6. Omicron BA.2 mt Spike protein/ACE2 complex (two and three ACE2 bound) (7XO7 and 7XO8)

OEU interactions on Omicron BA.2 mt Spike protein/ACE2 complex with two and three ACE2 bound (7xo7) include M731 (polar, 3.7 Å). K947 (H–bond, 2.4 Å), D950 (H–bond, 2.1 Å), H954 (π–π displaced, 3.8 Å), Q957 (H–bond, 3.2, 3.3 Å, π–polar, 3.3 Å), Q1010 (polar, 3.0 Å), R1014 (Hph, 3.3 Å), E1017 (2.7 Å), and I1018 (Hph, 3.7 Å) residues of protomer a (purple blue), and Q762 (3.9 Å), R765 (π–polar, 2.4, 2.9 Å, π–alkyl, 3.6 Å), A766 (polar 3.3 Å), G769, E773 (polar, 3.7 Å), V951 (Hph, 3.8 Å), R1019 (polar 3.0 Å), I312 (π–polar, 3.5 Å) residues of protomer c (purple).

On the other hand, OC is stabilized at the interface between the S1/S2 furin cleavage site, the fusion peptide (FP) and the S2′ domains. OC is positioned in its binding pocket with the incorporation of I312 (polar, 2.9 Å, Hph, 3.5 Å), Q314 (H–bond, 3.5 Å, π–polar, 3.2 Å, and π–alkyl, 2.9 Å), S596 (H–bond, 3.4 Å), Q613 (H–bond, 2.6 Å), P665 (H–bond, 2.3 Å), and I666 (H–bond, 3.9 Å) residues of protomer a (purple blue), and additionally T768 (polar and π–polar, 3.5 Å), V772 (polar, 3.2 Å), and D775 (H–bond, 3.8 Å) residues of protomer a (purple blue).

S2.7. Wt full–length S protein's RBD/ACE2 complex (from 6M17)

The anchorage of OEU is facilitated by the formation of hydrogen bond, hydrophobic (alkyl–alkyl type), polar, π–polar, mixed π–type hydrophobic contacts (π–alkyl type), and π–cation and π–anion

electrostatic interactions. Amino acid residues participating in these interactions contributing to binding affinity between RBD and ACE2 include: Asp (D30) (H–bond, 3.6, 3.7 Å), His (H34) (π–polar, 2.5 Å), Glu (E35) (H–bond, 2.5 Å), Glu (E37) (H–bond, 2.5, 3.5 Å), and Asp (D38) (π–anion, 2.4 Å), (belonging to ACE2 receptor), and: Arg (R402) (H–bond, 2.1, 3.2, and 3.6 Å), Arg (R403) (H–bond, 2.1, 3.2 Å), Lys (K417) (polar, 2.4 Å), Tyr (Y453) (H–bond, 3.9 Å), Gln (Q493) (H–bond, 2.3 Å), Ser (S494) (H–bond, 2.0, 3.0 Å), Tyr (Y495) (polar, 3.1 Å), and Tyr (Y505) (π–polar, 2.0, 3.5 Å, π–alkyl, 3.0 Å), (belonging to RBD domain).

Likewise, the OC interactions with ACE2 predicted to be: His (H34) (π–polar, 3.3 Å), Glu (E37) (π–anion, 3.2 Å, Hph, 3.3 Å), Asp (D38) (H–bond, 2.6 Å), Lys (K353) (H–bond, 2.6 Å, Hph, 3.1 Å) residues (belonging to ACE2 receptor), and: Arg (R403) (H–bond, 3.3 Å, π–cation, 2.4 Å), Tyr (Y449) (H–bond, 2.6 Å, π–polar, 3.6 Å), Gln (Q493) (polar, 3.8 Å), Tyr (Y495) (H–bond, 2.8 Å), Gln (Q498) (H–bond, 3.5 Å), Asn (N501) (H–bond, 2.4 Å), and Tyr (Y505) (π–π displaced, 3.0, 3.2 Å, H–bond, 3.1 Å) residues (belonging to RBD domain).

S2.8. Wt S proteins' RBD/ACE2 complex (6VW1)

OEU makes numerous contacts including the residues T276/Cγ2 (Hph, 2.8 Å), D292/Oδ2 (H-bond, 2.8 Å), M366/Sδ (polar, 3.6 Å), D367/O, Oδ1, Oδ2 (H–bond, 2.8, 3.4, 3.5 Å), L370/Cδ1 (π–alkyl, 3.0 Å), T371/Cγ2 (π–alkyl, 3.4 Å, π–polar, 3.8 Å), **E406**/Oε2 (π–anion, 2.5 Å), **S409**/O, Oγ (polar, 3.6 Å, π–polar, 3.2 Å), L410/Cδ1 (Hph, 3.9 Å), **K441**/Nζ (H–bond, 3.2 Å, Hph, 2.9 Å), **Q442**/Nε2, Oε1 (H–bond, 3.4 Å, polar, 2.4 Å), and **T445**/Oγ1 (polar, 3.5 Å).

OC is anchored in the same binding pocket with OEU, with the inclusion of residues **E406**/Oε2 (π–anion, 2.4, 3.2 Å), **S409**/Oγ (H–bond, 3.7 Å, π–polar, 2.9 Å), A413/Cβ (Hph, 3.4 Å), F438/O, Cδ1 (H–bond, 2.4 Å, π–alkyl, 3.7 Å), **Q442**/Nε2 (H–bond, 3.2 Å), **K441**/Nζ, O (H–bond, 2.0 Å, polar, 3.5 Å), **Q442**/O, Oε1, Cγ (H–bond, 3.9 Å , polar, 2.7, 2.1 Å, Hph, 2.3 Å), **T445**/Oγ1 (H–bond, 3.65 Å, Hph, 3.2 Å), and I446/Cγ1 (Hph, 3.3 Å). Common binding contacts between OEU and OC are indicated in boldface type.

S2.9. Delta and Kappa S proteins' RBD/ACE2 complex (7V8B and 7V87)

The 3,4–dihydroxyphenethyl ester moiety of OEU is H–bond connected to Y495/O (3.2 Å) and S494/NH, Oγ (2.8 Å, 3.4 Å) of RBD, H34/Nε2 (2.5 Å) of ACE2. Additionally, a π–π displaced contact (2.9–3.3 Å) is observed between the aromatic rings of OEU and H34. π–π sandwich between 3,4–dihydroxyphenyl ring of OEU and the aromatic ring of Y449 (2.9-3.8 Å). 3,4–dihydroxyphenyl ring of OEU observed in π–polar contact with S494/O (2.6 Å) of RBD and π–anion contact with D38 (2.6 Å) of ACE2. Esteric O of 3,4–dihydroxyphenyl ring of OEU is polar connected to H34/O (2.3 Å) and E37/Oε1 (3.3 Å) of ACE2 and also its phenethyl ester moiety is interacted via Hph bond with E37/Cβ (3.1 Å) and D38/Cβ (2.8 Å). Methyl ester's group O of OEU is hydrogen bonded to K353/NζH (3.4 Å) of ACE2 and G496/NH (2.9 Å) of RBD. Ethylidene moiety's C atom is π–alkyl connected to Y505 (3.1 Å) of RBD. Between beta–D–glucoside moiety O atoms in polar (P) contact with R403/Nη1 (3.4 Å) and H–bond with E406/Oε2 (2.9 Å), R454/O (3.6 Å) and Y495/OH (2.8 Å) of RBD. Furthermore, the pyran-O and C atoms of OEU is π–polar (2.4 Å) and π–alkyl (3.5 Å) contacted to Y495 of RBD. Also, Hph contacts are observed between the beta–D–glucoside moiety O atoms of OEU with K417/Cγ (2.6 Å), L455/Cδ1 (2.6 Å) and π–alkyl contacts with Y453 ring (2.7 Å).

OC is stabilized at the interface between RBD domain and ACE2 protein receptor of Kappa (7v87) variant with the following residues: N33/Nδ2, Oδ1 (H–bond, 2.0, 2.9 Å), H34 (π–π dis-placed (offset), 2.7 Å), E37/Oε1, Oε2 (H-bond, 2.3, 2.4 Å), N388/O (H–bond, 3.7 Å), P389/Cα (polar, 3.3 Å), R393/Nη2 (H–bond, 3.7 Å) of ACE2, and E406/Oε1 (H–bond), Y453/OH (π–polar, 3.3 Å), and Y453 (π–π displaced (offset), 3.8 Å) of RBD.

S2.10. Omicron BA.1 and BA.2 mt S proteins' RBD/ACE2 complex (PDB ascension N's 7WPB and 7XO9)

The stabilization of OEU to ACE2 human receptor involves the following binding interactions: The 3,4-dihydroxyphenethyl ester moiety of OEU is π–alkyl hydrophobic (Hph) bonded to L95/Cδ2,

Cδ1 (2.9 Å, 3.4 Å) and V209/Cγ1 (3.7 Å), H–bond connected to K562/O (3.0 Å), and additionally interacted *via* π–polar contact to E564/O (3.9 Å) and E208 (4.1 Å). The ethyl's group C atom of 3,4-dihydroxyphenethyl ester moiety is π–alkyl bonded to W566 (3.9 Å). The carboxylic acid moiety's O of the carboxymethyl group is polar contacted to A396/O (3.0 Å). The ethylidene moiety of OEU is connected to D206 through Hb with Oδ2 (3.0 Å), polar contact with Oδ1 (3.0 Å), and Hph contact to Cγ (3.4 Å). Also, additional stabilization is achieved through polar contacts of E208/O and N with the carboxymethyl group of 3,4-dihydro-2H-pyran-5-carboxylic acid moiety of OEU (3.1 Å and 3.9 Å), the C atom in *α* position to ethylidene moiety is Hph bonded to K562/Cε (2.9 Å). Furthermore, the beta-D-glucoside moiety (by its O atoms) is found to be attached to K562/Nζ *via* electrostatic contacts (2.4 Å, 2.7 Å, and 3.1 Å), A99/O *via* H–bond (2.1 Å, 3.0 Å), and to L391/O, Cδ2, Cα *via* H–bond and Hph contacts (4.0 Å, 2.7 Å, and 3.5 Å).

The stabilization of OC to ACE2 human receptor is achieved through the following binding interactions: H–bond contacts between the phenolic O of 2-(p-hydroxyphenyl)ethyl ester moiety and the E564/O (2.7 Å) and K562/O (3.3 Å), 2-(p-hydroxyphenyl)ethyl ester moiety *via* π-polar contacts to K562/O (3.2 Å), A396 (2.6 Å) and π–alkyl hydrophobic contact to K562/Cγ (3.4 Å). Additional contacts of OC involve H–bond and Hph binding to K562/Nζ, Cε (3.6 Å, 3.6 Å), polar of enol aldehyde group O to E208/O (3.9 Å) and Q98/Oε1, Nε2 (3.1 Å, 3.6 Å), as well as polar and H–bond of the other aldehyde group O to Q98/O (3.4 Å) and A99/NH (3.8 Å), respectively. The ethylidene moiety is also Hph contacted to L95/Cδ2 (3.4 Å). The aldehyde group O is also found in H–bond and polar contacts to Q102/Nε2 (2.4 Å) and Oε1 (3.2 Å), respectively. Common binding interactions between OEU and OC were revealed to be residues Glu (E564), Lys (K562), Ala (A396), Glu (E208), Leu (L95) and Ala (A99).

S2.11. Omicron BA.2 mt S proteins' RBD/ACE2 complex (ascension Nr 7ZF7)

The 3,4–dihydroxyphenethyl ester moiety of OEU is H–bond connected to Y495/O (3.2 Å) and the stabilization of OEU to ACE2 human receptor involves the following binding interactions:

Binding interactions of OEU with ACE2 revealed to be: T276/Cγ2 (Hph, 2.8 Å), N290/Oδ1 (H–bond, 2.1 Å), I291/NH, Cδ1 (H–bond, 3.5 Å, polar, 3.5 Å), M366/Cε (Hph, 3.4 Å), D367/Oδ2 (polar, 3.4 Å), A413/O (H–bond, 3.5 Å), P415/Cγ (po-lar, 3.4 Å), T434/O, Cβ (H–bond, 2.3 Å, polar, 3.3 Å), F438 (π–polar, 3.5 Å, π–alkyl, 3.0–3.6 Å, Hph, 3.6 Å), K441/Cγ, O (π–alkyl, 2.8 Å, π–polar, 3.1 Å), and N442/Oε1, N (H–bond, 2.2, 2.5 Å, π–polar, 2.8, 3.8 Å). The interacting residues belonging to the ACE2 receptor are T276, N290, I291, M366, D367, A413, P415, T434, F438, K441, and N442.

Binding interactions of OC with ACE2 revealed to be: L95/Cδ2 (polar, 2.6 Å), N98/Oε1 (H–bond, 3.7 Å), A99/NH (H–bond, 3.0 Å), N102/Nε2 (H–bond, 3.8 Å), D206/O (H-bond, 2.1 Å), Y207/N (H–bond, 3.6 Å), E208/O (polar, 2.8 Å), A396/O (π–polar, 2.6 Å), N397/Cα (π–alkyl, 3.9 Å). K562/Nζ, O (salt bridge, 3.0 Å, polar, 2.5 Å), E564/O (polar, 3.7 Å), and W566 (O–π–polar, 2.8, 3.6 Å, π–π T–shaped, 3.3 Å, N–3.6 Å).

S2.12. N501Y mt RBD in complex with COVOX–269 Fab (7NEG)

The following interactions of OEU with the N501Y mt RBD in complex with COVOX–269 Fab were observed: a) with RBD: K417/Nζ (H–bond, 3.1 Å), R403/Nη1 (H–bond, 2.8, 3.1 Å, salt bridge, 3.4 Å), D405/O (H–bond, 2.2 Å), E406/Cγ (Hph, 3.5 Å), R408/Nε, Cδ (H–bond, 3.7 Å, Hph, 3.7 Å), Q409/Nε2, Cγ (H–bond, 3.1 Å, Hph, 3.3 Å), and Q414/Oε1 (polar, 3.5 Å), b) with H chain of Fab–269 (hot pink cartoon): Y52/OH (H–bond, 2.7 Å), F58/Cε1 (π–alkyl, 3.5 Å, π–polar, 3.3 Å), Y59/O (π–polar, 3.8 Å), D61/Oδ1 (p–anion, 3.3 Å), and K64/Nζ (π–cation, 3.7 Å), and c) with L chain of Fab–269 (deep purple cartoon): N92/Oδ1, O (H–bond, 2.4 Å, polar, 3.7 Å), Y94/O (polar, 3.5 Å), P95/O, Cγ (H–bond, 2.6 Å, Hph, 2.5 Å, π–polar, 2.6 Å), and A96/Cβ (polar, 2.8 Å).

Binding interactions of OC with RBD revealed to be: N360/N, Nδ2 (H–bond, 2.6, 3.1, 3.3 Å), C361/NH, C (H–bond, 2.6, Å, Hph, 3.61 Å), A363/N, Cβ (π–polar, 3.2 Å, π–alkyl, 3.7 Å), Y365 (π–π displaced, 4.0–4.2 Å), N388/O (H–bond, 2.5 Å), L390/O (H–bond, 2.8 Å), F392/O, NH (H–bond, 2.1, 2.7 Å), and V395/Cγ2 (Hph, 3.9 Å). N360, C361, and A363 residues belong to loop between β1/*α*3, N388 to loop between β2/β3, L390 and F392 to the part of loop connecting β2 with β3, while Y365 to the *α*3 helix and V395 is the first residue of strand β3.

References

1. Casalino, L.; Gaieb, Z.; Goldsmith, J. A.; Hjorth, C. K.; Dommer, A. C.; Harbison, A. M.; Fogarty, C. A.; Barros, E. P.; Taylor, B. C.; McLellan, J. S.; Fadda, E.; Amaro, R. E. Beyond Shielding: The Roles of Glycans in SARS-CoV-2 Spike Protein. *ACS Cent. Sci*. **2020**, ASAP. doi: 10.1021/acscentsci.0c01056.
2. Wrapp, D.; Wang, N.; Corbett, K. S.; Goldsmith, J. A.; Hsieh, C.-L.; Abiona, O.; Graham, B. S.; McLellan, J. S. Cryo-EM Structure of the 2019-NCoV Spike in the Prefusion Conformation. *Science* **2020**, 367, 1260−1263, doi: 10.1126/science.abb2507.
3. Walls, A. C.; Park, Y.-J.; Tortorici, M. A.; Wall, A.; McGuire, A. T.; Veesler, D. Structure, Function, and Antigenicity of the SARS-CoV-2 Spike Glycoprotein. *Cell* **2020**, 181, 281, doi: 10.1016/j.cell.2020.02.058.
4. Benton, D.J.; Wrobel. A.G.; Xu, P.; Roustan, C.; Martin, S.R.; Rosenthal, P.B.; Skehel, J.J.; Gamblin, S.J. Receptor binding and priming of the spike protein of SARS-CoV-2 for membrane fusion. *Nature* **2020**, 588(7837), 327–330. doi: 10.1038/s41586-020-2772-0.
5. Gobeil, S.M.; Janowska, K.; McDowell, S.; Mansouri, K.; Parks, R.; et al. D614G mutation alters SARS-CoV-2 Spike conformation and enhances protease cleavage at the S1/S2 junction. *Cell Rep*. **2021**, 34(2), 108630. doi: 10.1016/j.celrep.2020.108630.
6. Mannar, D.; Saville, J.W.; Sun, Z.; Zhu, X.; Marti, M.M.; Srivastava, S.S.; Berezuk, A.M.; Zhou, S.; Tuttle, K.S.; Sobolewski, M.D.; Kim, A.; Treat, B.R.; Da Silva Castanha, P.M.; Jacobs, J.L.; Barratt-Boyes, S.M.; Mellors, J.W.; Dimitrov, D.S.; Li, W.; Subramaniam, S. SARS-CoV-2 variants of concern: spike protein mutational analysis and epitope for broad neutralization. *Nat. Commun.* **2022**, 13(1), 4696. doi: 10.1038/s41467-022-32262-8.
7. Yang, T.J., Yu, P.Y., Chang, Y.C., Hsu, S.T.D. Local refinement of SARS-CoV-2 S-Kappa variant (B.1.617.1) RBD and Angiotensin-converting enzyme 2 (ACE2) ectodomain, *to be published*.
8. Ye, G.; Liu, B.; Li, F. Cryo-EM structure of a SARS-CoV-2 omicron spike protein ectodomain *Nat. Commun.* **2022**, 13, 1214. doi: 10.1038/s41467-022-28882-9.
9. Cao, Y.; Yisimayi, A.; Jian, F.; Song, W.; Xiao, T.; et al. BA.2.12.1, BA.4 and BA.5 escape antibodies elicited by Omicron infection. *Nature* **2022**, 608(7923), 593–602. doi: 10.1038/s41586-022-04980-y.
10. Supasa, P.; Zhou, D.; Dejnirattisai, W.; Liu, C.; et al. Reduced neutralization of SARS-CoV-2 B.1.1.7 variant by convalescent and vaccine sera. *Cell* **2021**, 184(8), 2201-2211.e7. doi: 10.1016/j.cell.2021.02.033.
11. Tuekprakhon, A.; Nutalai, R.; Dijokaite-Guraliuc, A.; Zhou, D.; Ginn, H.M.; et al. Antibody escape of SARS-CoV-2 Omicron BA.4 and BA.5 from vaccine and BA.1 serum. *Cell* **2022**, 185(14), 2422–2433.e13. doi: 10.1016/j.cell.2022.06.005.
12. Xiao, T.; Lu, J.; Zhang, J.; Johnson, R.I.; McKay, L.G.A.; Storm, N. et al. A trimeric human angiotensin-converting enzyme 2 as an anti-SARS-CoV-2 agent. *Nat. Struct. Mol. Biol.* **2021**, 28, 202–209. doi: 10.1038/s41594-020-00549-3
13. Xu, Y.; Wu, C.; Cao, X.; Gu, C.; Liu, H.; Jiang, M.; Wang, X.; Yuan, Q.; Wu, K.; Liu, J.; Wang, D.; He, X.; Wang, X.; Deng, S.J.; Xu, H.E.;Yin, W. Structural and biochemical mechanism for increased infectivity and immune evasion of Omicron BA.2 variant compared to BA.1 and their possible mouse origins. *Cell Res.* **2022**, 32(7), 609–620. doi: 10.1038/s41422-022-00672-4.
14. Shang, J.; Ye, G.; Shi, K.; Wan, Y.; Luo, C.; Aihara, H.; Geng, Q.; Auerbach, A.; Li, F. Structural basis of receptor recognition by SARS-CoV-2. *Nature* **2020**, 581(7807), 221–224. doi: 10.1038/s41586-020-2179-y.
15. Yin, W.; Xu, Y.; Xu, P.; Cao, X.; Wu, C.; Gu, C.; He, X.; Wang, X.; et al. Structures of the Omicron spike trimer with ACE2 and an anti-Omicron antibody. *Science* **2022**, 375 (6584), 1048–1053. doi: 10.1126/science.abn8863.
16. Nutalai, R.; Zhou, D.; Tuekprakhon, A.; Ginn, H.M.; et al. Potent cross-reactive antibodies following Omicron breakthrough in vaccinees. *Cell* **2022**, 185(12), 2116–2131.e18. doi: 10.1016/j.cell.2022.05.014.
17. Berman, H.M.; Westbrook, J.; Feng, Z.; Gilliland, G.; Bhat, T.N.; Weissig, H.; Shindyalov, I.N.; Bourne, P.E. The Protein Data Bank. *Nucleic Acids Res.* **2000**, 28, 235–242. doi: 10.1093/nar/28.1.235.
18. Berman, H.M.; Henrick, K.; Nakamura, H. Announcing the worldwide Protein Data Bank. *Nature Struct. Mol. Biol.* **2003**, 10, 980. doi: 10.1038/nsb1203-980.
19. Bernstein, F.C.; Koetzle, T.F.; Williams, G.J.; Meyer, E.E.; et al. The Protein Data Bank: a computer-based archival file for macromolecular structures. *J. Mol. Biol.* **1977**, 112(3), 535–542. doi: 10.1016/s0022-2836(77)80200-3.
20. Fosgerau, K.; Hoffmann, T. Peptide therapeutics: current status and future directions. *Drug Discov. Today* **2015**, 20(1), 122–128. doi: 10.1016/j.drudis.2014.10.003.
21. Shivakumar, D.; Williams, J.; Wu, Y.; Damm, W.; Shelley, J.; Sherman, W. Prediction of absolute solvation free energies using Molecular Dynamics free energy perturbation and the OPLS force field. *J. Chem. Theory Comput.* **2010**, 6(5), 1509–1519. doi: 10.1021/ct900587b.

22. Sastry, G.M.; Adzhigirey, M.; Day, T.; Annabhimoju, R.; Sherman, W. Protein and ligand preparation: parameters, protocols, and influence on virtual screening enrichments. *J. Comput. Aided Mol. Des.* **2013**, 27(3) 221–234. doi: 10.1007/s10822-013-9644-8.
23. Friesner, R.A.; Murphy, R.B.; Repasky, M.P.; Frye, L.L; Greenwood, J.R.; Halgren, T.A.; Sanschagrin, P.C.; Mainz, D.T. Extra precision glide: docking and scoring incorporating a model of hydrophobic enclosure for protein-ligand complexes. *J. Med. Chem.* **2006**, 49(21), 6177–6196. doi: 10.1021/jm051256o.
24. Halgren, T.A.; Murphy, R.B.; Friesner, R.A.; Beard, H.S.; Frye, L.L.; Pollard, W.T.; Banks, J.L. Glide: a new approach for rapid, accurate docking and scoring. 2. Enrichment factors in database screening. *J. Med. Chem.* **2004**, 47(7), 1750–1759. doi: 10.1021/jm030644s.
25. Jacobson, M.P.; Pincus, D.L.; Rapp, C.S.; Day, T.J.F.; Honig, B.; Shaw, D.E.; Friesner, R.A. A hierarchical approach to all-atom protein loop prediction. *Proteins* **2004**, 55(2), 351–367. doi: 10.1002/prot.10613.
26. Jacobson, M.P.; Friesner, R.A.; Xiang, Z.; Honig, B. On the role of the crystal environment in determining protein side-chain conformations. *J. Mol. Biol.* **2002**, 320(3), 597–608. doi: 10.1016/s0022-2836(02)00470-9.
27. Halgren, T. New method for fast and accurate binding-site identification and analysis. *Chem. Biol. Drug Des.* **2007**, 69(2), 146–148. doi: 10.1111/j.1747-0285.2007.00483.x.
28. Halgren, T. Identifying and characterizing binding sites and assessing druggability. *J. Chem. Inf. Model.* **2009**, 49(2), 377–389. doi: 10.1021/ci800324m.
29. Friesner, R.A.; Banks, J.L.; Murphy, R.B.; Halgren, T.A.; Klicic, J.J.; Mainz, D.T.; et al. Glide: A New Approach for Rapid, Accurate Docking and Scoring. 1. Method and Assessment of Docking Accuracy *J. Med. Chem.* **2004**, 47(7), 1739–1749. doi: 10.1021/jm0306430.
30. Farid, R.; Day, T.; Friesner, R.A.; Pearlstein, R.A. New insights about HERG blockade obtained from protein modeling, potential energy mapping, and docking studies. *Bioorg. Med. Chem.* **2006**, 14(9), 3160–3173. doi: 10.1016/j.bmc.2005.12.032.
31. Sherman, W.; Day, T.; Jacobson, M.P.; Friesner, R.A.; Farid, R. Novel procedure for modeling ligand/receptor induced fit effects. *J. Med. Chem.* **2006**, 49(2), 534–553. doi: 10.1021/jm050540c.
32. Sherman, W.; Beard, H.S.; Farid, R. Use of an induced fit receptor structure in virtual screening. *Chem. Biol. Drug Des.* **2006**, 67(1), 83-4. doi: 10.1111/j.1747-0285.2005.00327.x.
33. Geromichalos, G.D.; Alifieris, C.E.; Geromichalou, E.G.; Trafalis, D.T. Overview on the current status of virtual high-throughput screening and combinatorial chemistry approaches in multi-target anticancer drug discovery; Part I. *J. BUON* **2016**, 21(4), 764–779. PMID: 27685895.
34. Bashford, D.; Case, D.A. Generalized born models of macromolecular solvation effects. *Annu. Rev. Phys. Chem.* **2000**, 51, 129–52. doi: 10.1146/annurev.physchem.51.1.129.
35. DeLano, W.L. The PyMol Molecular Graphics System 0.99, DeLano Scientific, San Carlos, CA, USA, 2006.
